# Personal risk factors associated with heat-related illness among new conscripts undergoing basic training in Thailand

**DOI:** 10.1371/journal.pone.0203428

**Published:** 2018-09-04

**Authors:** Rudchanu Nutong, Mathirut Mungthin, Panadda Hatthachote, Supak Ukritchon, Worarachanee Imjaijit, Pimrapat Tengtrakulcharoen, Suthee Panichkul, Panwadee Putwatana, Wonnapha Prapaipanich, Ram Rangsin

**Affiliations:** 1 Ramathibodi School of Nursing, Faculty of Medicine Ramathibodi Hospital, Mahidol University, Bangkok, Thailand; 2 Department of Pharmacology, Phramongkutklao College of Medicine, Bangkok, Thailand; 3 Department of Physiology, Phramongkutklao College of Medicine, Bangkok, Thailand; 4 Office of Research and Development, Phramongkutklao College of Medicine, Bangkok, Thailand; 5 Department of Military and Community Medicine, Phramongkutklao College of Medicine, Bangkok, Thailand; Weill Cornell Medical College in Qatar, QATAR

## Abstract

Cases of exertional heat stroke have been reported every year during basic training for Royal Thai Army (RTA) conscripts. Prevention is an important strategy to reduce the incidence of heat-related illnesses. We conducted a study to identify potential indicators for the prevention and monitoring of heat-related illnesses among military conscripts undergoing basic training in Thailand. All newly inducted RTA conscripts in 5 basic training units in 5 regions in Thailand were invited to participate in a prospective cohort study from May 1 to July 9, 2013. The incidence rate of heat-related illnesses and the incidence rate ratio (IRR) based on a Poisson regression model were used to identify the independent factors associated with heat-related illnesses, daily tympanic (body) temperatures higher than 37.5°C, >3% decreases in body weight in one day, and the production of dark brown urine. Eight hundred and nine men aged 21.4 (±1.13) years were enrolled in this study. The prevalence of a body mass index (BMI) ≥30 kg/m^2^ was 5.5%. During the study period, 53 subjects (6.6%) representing 3.41/100 person-months (95% confidence interval (CI), 2.55–4.23) developed heat-related illnesses (excluding heat rash), and no subjects experienced heat stroke. The incidence rates of a daily tympanic temperature >37.5°C at least once, body weight loss of >3% per day, and the production of dark brown urine at least once were 8.27/100 person-months (95% CI, 7.69–8.93), 47.91/100 person-months (95% CI, 44.22–51.58), and 682.11/100 person-months (95% CI, 635.49–728.52), respectively. The sole identified independent factor related to the incidence of heat-related illnesses was a BMI ≥30 kg/m^2^ (adjusted IRR = 2.66, 95% CI, 1.01–7.03). In conclusion, a high BMI was associated with heat-related illnesses among conscripts undergoing basic training in Thailand. Daily monitoring of heat-related illnesses, body temperature, body weight and urine color in each new conscript during basic military training was feasible.

## Introduction

Heat-related illnesses, including heat stroke, are major public health problems that have particular relevance to military operations. Heat-related illnesses comprise a group of symptoms that occur in response to heat accumulation in the body caused by exercise or work in a hot and humid environment for a long period of time until the body is unable to regulate its temperature. As a result, organ systems fail as symptoms increase in severity from mild to severe, eventually leading to death if allowed to progress untreated [1–4]. The most severe form of heat-related illness is heat stroke, in which the case fatality rate is high, up to 50%. The treatment outcomes of heat stroke are mainly related to the duration and intensity of hyperthermia. Therefore, early detection and timely, effective treatment, including early rapid body cooling, should be applied to heat stroke patients [5]. Military conscripts and athletes in training who have not undergone heat acclimatization are at risk for heat-related illness [6]. Risk factors for heat-related illness include both personal and environmental variables [2,7,8]. Environmental factors include hot weather and high humidity [9–14]. Personal factors include physical condition, ethnicity, genetics and behavior; because personal factors differ among individuals, people who are in the same environment can experience the effects of heat-related illnesses differently [15,16]. Studies in athletes have found that risk factors for heat-related illness comprise external factors, including the temperature, humidity, level of activity and clothing worn, in addition to internal factors, including age at first alcohol use, dehydration, obesity, current illness, history of heat-related illness, medication, inadequate heat acclimatization and insufficient rest and sleep at night [15,17–21].

The key principle in preventing heat stroke in new conscripts is the control of risk factors [22]. Early detection of risk factors prior to an episode of heat-related illness should be a high priority. We would like to identify relatively simple and noninvasive measurements that could fit into the conscripts’ daily schedule. Daily measurements of body temperature, body weight and urine color for all new conscripts during basic training may be practical for the prevention of heat-related illnesses. Therefore, we performed a follow-up study to identify the feasibility of collecting daily measurements to assess factors associated with heat-related illness, including a daily tympanic (body) temperature greater than 37.5°C, body weight loss of more than 3% in one day, and the production of dark brown urine, during the 10-week military training period.

## Methods

### Population and sampling

Each year, 60,000–80,000 men who are approximately 21 years of age are conscripted into the Royal Thai Army (RTA). Induction occurs in either May or November and begins a two-year duration of military service. Heat-related illnesses, including heat stroke, have been reported every year during the 10-week basic military training. Prior to 2012, the RTA implemented a heat-related illness prevention program during basic training, using the environmental temperature and humidity to guide the duration of training and rest periods and the amount of water consumption recommended during each hour of training. No personal factors were considered at that time. In 2012, the RTA began to recognize the contribution of the personal factors of each new conscript to the development of heat-related illnesses. Since then, the RTA heat-related illness prevention program has included the screening of high-risk individuals, e.g., subjects with a high body mass index (BMI), acute respiratory or gastrointestinal illnesses, or current use of illicit drugs. Those who are at high risk are trained with special precautions and at a lower training intensity than the other conscripts. In 2013, 21 new conscripts were diagnosed with heat stroke, including 2 fatalities, during basic training. That high number of cases led the RTA Medical Department to strengthen the RTA prevention and control program for heat-related illnesses during basic training, which improved the surveillance program and treatment protocol for heat-related illness [23].

We selected 5 basic training units of the RTA from 5 regions of the country, including the North (Chiang Mai), Northeast (Ubon Ratchathani), Central (Lop Buri), South (Songkhla) and Bangkok. All newly inducted conscripts as of May 2013 were invited to participate in the study.

The daily schedule of RTA basic military training on the 5 working days of the week includes waking up (5:30 AM), morning physical training (6:00 AM-7:00 AM), 4 morning 50-minute training sessions (8:00 AM-12:00 PM), 3 afternoon 50-minute training sessions (1:00 PM-4:00 PM), afternoon physical training (4:00 PM-6:00 PM) and retiring to bed (9:00 PM).

The 10-week basic training program is divided into 4 phases. During Phase 1 (Weeks 1–2), conscripts are trained under a “heat acclimatization” protocol, which gradually increases in physical intensity based on individual baseline fitness, in addition to drill and ceremony training. Phase 2 (Weeks 3–5) includes Combat Fundamentals and unarmed combat training. Phase 3 (Weeks 6–8) is for armed combat training and tactical training. The last phase, Phase 4 (Weeks 9–10), consists of field training exercises and evaluations.

Since 2017, the RTA has implemented an additional heat acclimatization protocol for those conscripts whose BMI is >30 kg/m^2^. This high-risk group partakes in a special physical exercise program designed with carefully increasing physical intensity. For example, there is no running during the first week. The protocol recommends starting the exercise schedule with 30 minutes of fast walking only in the morning during the first 3 days and increasing the duration to 45 minutes of fast walking in the morning by the end of the first week.

### Data collection

Data from all conscripts were collected through standardized questionnaires after the participants signed the consent form; the data included unit information, personal information, environmental conditions and daily activity information. Wet- and dry-bulb temperatures in the training areas were measured at 7:00 AM, 11:00 AM, 1:00 PM, and 4:00 PM to assess the air temperature and relative humidity of the environment. The relative humidity was obtained from a standard relative humidity table [23] using the difference between the wet-bulb and dry-bulb temperatures. The subjects were followed for approximately 10 weeks after entry.

Baseline personal information was collected using a standardized questionnaire within the first week of the training. During the 10-week training period, the conscripts’ tympanic temperature and body weight were measured daily before bed time using a Citizen CT 810 digital ear thermometer (Citizen Holdings Co., Tokyo, Japan) and an Oxygen Cycle digital body weight scale (KP Kamolkij Intertrade Co., Ltd., Bangkok, Thailand), respectively. Medical personnel in each training unit were trained to perform the measurements using a digital thermometer and digital weight scale under the same study protocol across all participating military units. Daily signs and symptoms were recorded by the supervisor of each unit. The recorded illnesses during basic training included fever, cold, sore throat, headache, diarrhea, heat rash, heat edema, heat cramps, heat syncope, heat exhaustion, and heat stroke. Every evening, the study participants were asked to provide urine samples in transparent containers to be assessed for urine color by the unit supervisors. The color of each participant’s urine sample was recorded daily. The urine colors were categorized into 4 groups: light yellow, yellow, dark yellow, and dark brown.

Heat-related illness incidents were defined as a diagnosis of one of the following illnesses based on the definitions provided by the RTA Medical Department [23], which corresponds to the Occupational Safety and Health Administration (OSHA) technical manual on heat stress [24] and the NIH Heat Stress Program [25]: heat stroke, heat exhaustion, heat syncope, heat cramps, heat edema, heat tetany and prickly heat or heat rash. The conscripts who were identified as having a heat-related illness were more closely monitored and assigned to a less intense training program; in some cases, participants were also sent to the medical clinic for further investigation. Among patients with heat-related illnesses, those who did not visit a hospital received a clinical diagnosis from trained medical personnel in the participating training units. Those who visited a hospital for heat-related illnesses were diagnosed accordingly by a physician.

### Data analysis

The mean and standard deviation are used to describe continuous data. Percentages are used to describe categorical data. Because prickly heat or heat rash are very mild forms of illness and the identification of these conditions in the training unit may not be reliable, we present heat-related illness outcomes both including and excluding prickly heat or heat rash. The incidence rates per 100 person-months of observation were calculated for 4 main outcomes: heat-related illnesses (excluding heat rash), tympanic temperature over 37.5°C [26–29], a decrease in body weight by more than 3% in one day [2,30,31], and production of dark brown urine [31–33]. The incidence of heat-related illness was ascertained by a history of experiencing any heat-related illness other than heat rash, i.e., heat edema, heat syncope, heat tetany, heat cramps, heat exhaustion, or heat stroke. The person-times of observation of those subjects who experienced heat-related illnesses (excluding heat rash) were censored at the date of the disease onset.

The incidence of tympanic temperature more than 37.5°C was defined as the number of days each subject exhibited a tympanic temperature over 37.5°C during the daily tympanic temperature measurement. A daily body weight loss of more than 3% in one day was obtained by comparing daily body weight with the body weight recorded on the previous day. The incidence of daily body weight loss of more than 3% was defined as the number of times each subject exhibited daily body weight loss of more than 3%. The incidence of dark urine was calculated based on the number of days that each participant produced dark brown urine.

A multivariate Poisson regression analysis was performed to obtain the adjusted incidence rate ratios (IR) and 95% CIs of the factors related to each of the 4 main outcomes. A p-value cut-off point of 0.05 was used in all statistical analyses. All analyses were conducted using STATA/MP for Windows version 12.1 (StataCorp LP, College Station, TX, USA).

### Ethics statement

The study protocol was reviewed and approved by the Institutional Review Board of the RTA Medical Department. Written informed consent was obtained from the participants after they read the information sheet. Health education on preventing heat-related illness was provided to all participants. Those participants who developed heat-related illnesses received medical care at military hospitals according to the standard of care in Thailand.

## Results

A total of 809 newly inducted RTA conscripts from 5 regions of Thailand were enrolled in this study. All of the enrolled conscripts were males aged 21.4 (±1.13) years. The majority of the conscripts were from training units in the Central region (38.9%). Nearly 60% of the enrolled participants presented a normal BMI, while 5.5% of the participants exhibited a BMI ≥30 km/m^2^. In terms of occupation prior to entering the RTA, the majority (58.2%) of the participants reported a work history consisting of indoor activities (i.e., unemployed, students, employees, and merchants), whereas 41.8% of the participants had worked in professions based on outdoor activities (i.e., farmers and laborers). At baseline, almost 70% of the subjects were current smokers. Nearly 40% of the participants reported engaging in regular exercise at least 3 days per week (Table 1).

**Table 1:** Demographic data.

Demographic data	Number of study participants (%)
**Geographical Army area (n = 809)**	
• Average (min.-max.) temperature and humidity (May-June 2013)	
Central (Lop Buri province)	315 (38.9)
• Temperature = 29.7°C (22.8–39.7°C)	
• Humidity = 70% (28–99%)	
Northern (Chiang Mai province)	163 (20.2)
• Temperature = 29.0°C (21.8–40.3°C)	
• Humidity = 67% (24–98%)	
Bangkok Metropolitan Region	136 (16.8)
• Temperature = 29.6°C (23.0–38.8°C)	
• Humidity = 76% (35–96%)	
North-Eastern (Ubon Ratchathani province)	129 (15.9)
• Temperature = 27.7°C (20.4–36.8°C)	
• Humidity = 71% (31–89%)	
Southern (Songkhla province)	66 (8.2)
• Temperature = 27.4°C (21.7–35.6°C)	
• Humidity = 83% (45–100%)	
**Age (yrs)–mean±SD**
**Body mass index (kg/m^2^) (n = 743)**	
<18.5	76 (10.2)
18.5–22.9	434 (58.5)
23.0–24.9	96 (12.9)
25.0–29.9	96 (12.9)
≥30.0	41 (5.5)
**Occupation prior to conscription (n = 765)**	
Unemployed	62 (8.1)
Student	115 (15.0)
Farmer (farming, gardening, or animals)	219 (28.6)
Employee	180 (23.5)
Laborer	74 (9.7)
Merchant	51 (6.7)
Others	64 (8.4)
**Occupation group prior to conscription(n = 701)**	
Indoor (unemployed, student, employee, or merchant)	408 (58.2)
Outdoor (Farmer or Laborer)	293 (41.8)
**Smoking in the past 12 months**	
Current smoker	516 (67.5)
Ex-smoker	56 (7.3)
Never smoked	193 (25.2)
**Exercise in the past 12 months (at least 3 days per week)**	
Yes	289 (39.8)
No	437 (60.2)

The mean environmental temperatures during the 10-week basic training period (May 1-July 9, 2013) at 7:00 AM, 11:00 AM, 1:00 PM, and 4:00 PM were 31.12°C (±2.84), 32.97°C (±2.89), 33.94°C (±2.96), and 33.03°C (±3.60), respectively. The mean relative humidity at 1:00 PM was 61.81% (±10.99%), and 42.2% of the training days exhibited a relative humidity above 70% at 1:00 PM. The mean relative humidity at 4:00 PM was 67.45% (±11.51%), and 27.0% of the training days exhibited a relative humidity above 70% at 4:00 PM.

Events related to heat-related illness were detected in 127 (15.7%) of the participants and could be categorized into prickly heat (n = 99, 12.2%), heat edema (n = 25, 3.1%), heat syncope (n = 14, 1.7%), heat tetany (n = 10, 1.2%), heat cramps (n = 10, 1.2%) and heat exhaustion (n = 1, 0.1%). There were no cases of heat stroke in this cohort. Because the diagnosis of prickly heat by the army medics in the training units may be subject to variability, we excluded prickly heat from the main outcome of interest. Fifty-three (6.6%) conscripts reported heat-related illnesses other than prickly heat, accounting for an incidence rate of 3.41 (95% CI, 2.55–4.23) per 100 person-months (Table 2).

**Table 2:** Heat-related outcomes among new conscripts during basic military training (n = 809).

Heat-related outcomes	Number (%)	No. of incidents	Incidence rate per 100 person-months (95% CI)
Heat-related illnesses without prickly heat	53 (6.6)	53	3.41 (2.55–4.23)
Tympanic body temperature greater than 37.5°C	104 (12.9)	136	8.27 (7.69–8.39)
>37.5°C-37.9°C	66 (8.2)	89	5.41 (4.94–5.87)
38.0°C-38.9°C	24 (3.0)	29	1.76 (1.55–1.97)
>39.0°C	14 (1.7)	18	1.09 (0.92–1.27)
Body weight loss of more than 3% in one day	463 (57.2)	788	47.91 (44.22–51.58)
Dark brown urine	675 (83.4)	11220	682.11 (635.49–728.52)

**Fig 1:**
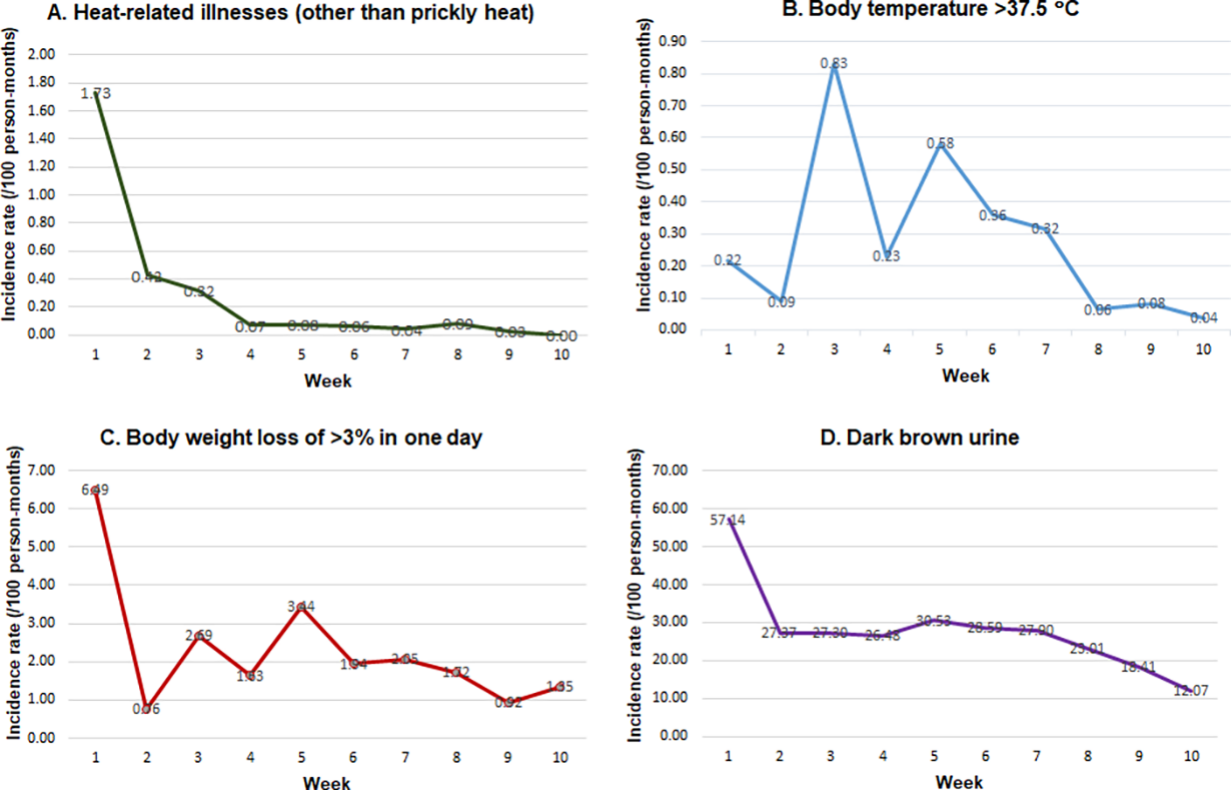
Incidence rates per 100 person-months of heat-related outcomes by week of basic training.

A total of 104 (12.9%) conscripts presented with a daily tympanic temperature above 37.5°C at some point, representing an incidence rate of 8.27 (95% CI, 7.69–8.39) per 100 person-months. During the study period, 463 participants (57.2%) experienced body weight loss of more than 3% in one day; the incidence rate was 47.91 (95% CI, 44.22–51.58). We also found that 675 (83.4%) participants had produced a dark urine sample, for an incidence rate of 682.11 (95% CI, 635.49–728.52) episodes per 100 person-months. Incidence rates per 100 person-months of heat-related illnesses (excluding prickly heat), body temperature greater than 37.5°C, body weight loss of more than 3% in one day, and dark brown urine by week of basic training are shown in Fig 1.

The univariate and multivariate analyses identifying personal risk factors for heat-related illnesses are shown in Table 3. The sole identified independent risk factor for heat-related illnesses (excluding prickly heat) was a BMI ≥30.0 kg/m^2^, with an IRR of 2.66 (95% CI, 1.01–7.03) compared to a normal BMI (18.5–22.9 kg/m^2^) after adjusting for smoking status. Other factors, including occupation prior to beginning training, smoking, and regular exercise in the past 12 months, were not associated with heat-related illnesses in our study.

**Table 3:** Personal risk factors associated with heat-related illness among new conscripts during basic military training.

Personal factors	No. of incidents		Incidence rate per 100 person-months	Univariate analysis		Multivariate analysis	
				IRR (95% CI)	p-value	IRR (95% CI)	p-value
**Occupation prior to conscriptions**							
Indoor	24		3.05	0.86 (0.47–1.55)	0.608		
Outdoor	20		3.56	1			
**Body mass index (kg/m**^**2**^**)**							
<18.5	7		4.86	1.78 (0.77–4.16)	0.180	1.85 (0.79–4.34)	0.156
18.5–22.9	23		2.72	1			
23.0–24.9	6		3.27	1.20 (0.49–2.95)	0.691	1.23 (0.50–3.05)	0.648
25.0–29.9	6		3.25	1.19 (0.49–2.93)	0.700	1.24 (0.50–3.05)	0.645
≥30.0	5		6.84	2.51 (0.95–6.60)	0.062	2.66 (1.01–7.03)	0.048
**Smoking in the past 12 months**							
Current smoker		34	3.43	1.61 (0.75–3.48)	0.224	1.58 (0.73–3.43)	0.245
Ex-smoker		5	4.75	2.23 (0.73–6.83)	0.158	2.14 (0.70–6.55)	0.183
Never smoked		8	2.12	1			
**Exercise in the past 12 months**							
No		24	2.84	0.71 (0.40–1.27)	0.251		
Yes		22	3.99	1			

Although there were no incidents of heat stroke in this study, we were able to identify risk factors associated with tympanic temperature higher than 37.5°C, a decrease in daily body weight by more than 3%, and dark brown urine in new conscripts undergoing basic military training.

The sole identified independent risk factor for a daily tympanic temperature of more than 37.5°C was a lack of regular exercise during the last 12 months prior to induction into the army (IRR = 1.62; 95% CI, 1.09–2.40) after adjusting for BMI (Table 4).

**Table 4:** Personal risk factors associated with a tympanic temperature greater than 37.5°C among new conscripts during basic military training.

Personal factors	No. of incidents	Incidence rate per 100 person-months	Univariate analysis		Multivariate analysis	
			IRR (95% CI)	p-value	IRR (95% CI)	p-value
**Occupation prior to conscriptions**						
Indoor	67	8.11	1.01 (0.69–1.46)	0.972		
Outdoor	48	8.05	1			
**Body mass index (kg/m**^**2**^**)**						
<18.5	11	7.12	0.86 (0.46–1.62)	0.641	0.91 (0.48–1.72)	0.762
18.5–22.9	27	8.27	1			
23.0–24.9	15	7.72	0.93 (0.54–1.63)	0.808	1.04 (0.60–1.83)	0.882
25.0–29.9	19	9.83	1.19 (0.72–1.97)	0.502	1.32 (0.79–2.19)	0.293
≥30.0	8	9.68	1.17 (0.56–2.43)	0.672	1.22 (0.58–2.53)	0.602
**Smoking in the past 12 months**						
Current smoker	84	8.02	0.98 (0.65–1.47)	0.907		
Ex-smoker	11	9.67	1.18 (0.59–2.34)	0.638		
Never smoked	32	8.21	1			
**Exercise in the past 12 months**						
No	84	9.50	1.51 (1.03–2.22)	0.037	1.62 (1.09–2.40)	0.017
Yes	37	6.29	1			

The independent risk factors for more than 3% weight loss in one day included an indoor occupation (IRR = 1.33; 95% CI, 1.12–1.58); BMI 23–24.9 kg/m^2^ (IRR = 1.30; 95% CI, 1.02–1.64), BMI 25.0–29.9 kg/m^2^ (IRR = 1.47; 95% CI, 1.17–1.83), or BMI ≥30.0 kg/m^2^ (IRR = 1.69; 95% CI, 1.23–2.33); and a history of not regularly exercising (IRR = 0.83; 95% CI, 0.71–0.98) (Table 5).

**Table 5:** Personal risk factors associated with body weight loss of more than 3% in one day among new conscripts during basic military training.

Personal factors	No. of incidents	Incidence rate per 100 person-months	Univariate analysis		Multivariate analysis	
			IRR (95% CI)	p-value	IRR (95% CI)	p-value
**Occupation prior to conscription**						
Indoor	446	54.00	1.33 (1.14–1.55)	<0.001	1.33 (1.12–1.58)	0.001
Outdoor	242	40.63				
**Body mass index (kg/m**^**2**^**)**						
<18.5	76	49.16	1.19 (0.93–1.53)	0.158	1.18 (0.91–1.53)	0.218
18.5–22.9	434	41.14	1			
23.0–24.9	96	54.06	1.31 (1.06–1.63)	0.014	1.30 (1.02–1.64)	0.030
25.0–29.9	96	59.52	1.45 (1.17–1.78)	0.001	1.47 (1.17–1.83)	0.001
≥30.0	41	64.17	1.56 (1.17–2.08)	0.003	1.69 (1.23–2.33)	0.001
**Smoking in the past 12 months**						
Current smoker	516	45.71	0.93 (0.79–1.10)	0.414		
Ex-smoker	56	58.95	1.20 (0.91–1.59)	0.194		
Never smoked	193	49.02	1			
**Exercise in the past 12 months**						
No	398	45.02	0.86 (0.74–1.00)	0.046	0.83 (0.71–0.98)	0.023
Yes	308	52.38	1			

Finally, the personal risk factors associated with dark brown urine production during basic training were BMI (<18.5 kg/m^2^, IRR = 1.23, 95% CI. 1.14–1.32; 23.0–24.9 kg/m^2^, IRR = 1.30, 95% CI, 1.22–1.38; 25.0–29.9 kg/m^2^, IRR = 1.65, 95% CI, 1.56–1.75; and ≥30.0 kg/m^2^, IRR = 1.71, 95% CI, 1.57–1.86), smoking status (current smoker IRR = 1.50, 95% CI, 1.42–1.58; ex-smoker, IRR = 1.17, 95% CI, 1.07–1.28), and a history of not regularly exercising (IRR = 1.10, 95% CI, 1.05–1.15) (Table 6).

**Table 6:** Personal risk factors associated with the production of dark brown urine by new conscripts during basic military training.

Personal risk factors	No. of incidents	Incidence rate per 100 person-months)	Univariate analysis		Multivariate analysis	
			IRR (95% CI)	p-value	IRR (95% CI)	p-value
**Occupation prior to conscriptions**						
Indoor	5630	681.67	1.05 (1.01–1.09)	0.022	1.00 (0.96–1.05)	0.945
Outdoor	3870	649.79	1			
**Body mass index (kg/m**^**2**^**)**						
<18.5	1191	771.24	1.29 (1.21–1.38)	<0.001	1.23 (1.14–1.32)	<0.001
18.5–22.9	5264	596.61	1			
23.0–24.9	1458	751.65	1.26 (1.19–1.33)	<0.001	1.30 (1.22–1.38)	<0.001
25.0–29.9	1890	978.19	1.64 (1.56–1.73)	<0.001	1.65 (1.56–1.75)	<0.001
≥30.0	873	1057.03	1.77 (1.65–1.90)	<0.001	1.71 (1.57–1.86)	<0.001
**Smoking in the past 12 months**						
Current smoker	996	763.04	1.42 (1.35–1.49)	<0.001	1.50 (1.42–1.58)	<0.001
Ex-smoker	747	657.34	1.22 (1.12–1.33)	<0.001	1.17 (1.07–1.28)	0.001
Never smoked	97	538.19	1			
**Exercise in the past 12 months**						
No	6474	732.32	1.11 (1.07–1.16)	<0.001	1.10 (1.05–1.15)	<0.001
Yes	3877	659.39	1			

## Discussion

In addition to identifying the risk factors associated with heat-related illnesses, another main purpose of this study was to examine indices for monitoring male conscripts undergoing basic military training in a hot, humid field setting from May to July in Thailand. We successfully followed a cohort of 809 young male conscripts during the 10-week training period using daily monitoring parameters to assess 1) heat-related illnesses, 2) tympanic temperature, 3) body weight, and 4) urine color. The proposed monitoring indices were feasible to implement in the field environment of basic military training.

The study population was composed of young Thai men who were relatively healthy by the time they were screened during the conscription process. We found that almost 60% of the participants had an occupational history of indoor activities, and almost 60% had a history of not regularly exercising prior to induction. Therefore, these subjects might not be as physically fit as the athletes included in prior studies related to heat-related illnesses.

Although there were no incidents of heat stroke in this study, our daily monitoring of heat-related illness revealed that several heat-related illnesses occurred during basic training, including heat exhaustion, which is a severe heat-related illness. The daily monitoring of illness may have prevented the development of heat stroke. The conscripts who were identified as having a heat-related illness were likely to be assigned to a less intense training program. When the physical activity of conscripts was reduced in a timely manner, the occurrence of exertional heat stroke in new conscripts was prevented.

When we analyzed the data to identify personal factors associated with heat-related illnesses, including heat exhaustion, heat syncope, heat cramps, heat edema, and heat tetany, we found that the only independent personal factor associated with heat-related illness was BMI. Participants with higher BMIs had a greater chance of developing heat-related illnesses; this finding was comparable to other reports [34–38]. In addition, a high BMI was associated with a decrease in daily body weight by more than 3% and dark brown urine. This finding has been reported in other military settings as well. US military personnel who were obese were 7.25 times more likely than normal-weight personnel to experience heat-related illness [4]. Soldiers with high BMIs who were undergoing basic training in the US were 2.1 times more likely to experience a heat-related illness than soldiers with low BMIs [38]. A trend of increasing BMI has been observed in recent decades, including in Thailand. The impact of BMI on developing heat-related illnesses during basic training should be emphasized. Alternative indicators of body composition specific to the Thai and Asian populations, e.g., body frame size, should be explored in future studies [39,40].

In addition, we studied the relationship between personal risk factors and personal indicators that may be proxy indicators of body heat accumulation and dehydration. These factors include known risk factors for heat-related illness, tympanic temperature higher than 37.5°C, a decrease in daily body weight by more than 3% and dark brown urine [41–43].

Several studies have reported that, after exercise under experimental conditions, the core body temperature remained elevated above resting levels for as long as 90 minutes [44]. However, in the present study, we measured each participant’s tympanic temperature in the evening before bedtime, around 7:00 PM-8:00 PM, which was 2–3 hours after the last session of afternoon exercise. Moreover, the daily tympanic temperature evaluation was performed in the training unit’s meeting room after a daily bath or shower session. Therefore, the temperature data in this study might not reflect the actual core body temperature at the time of training or exercise. However, measuring the new conscripts’ tympanic temperature in the evening before bedtime could facilitate the identification of subjects with a relatively high tympanic temperature of more than 37.5°C and those subjects who have a fever; these subjects could then be closely evaluated before starting training on the following day. Through this strategy of daily tympanic temperature measurement, a conscript who had a fever would be identified early and transferred for further medical evaluation to identify the cause of the fever, such as infection or other disease [1,45]. When we evaluated the association between the personal risk factors and daily tympanic temperature higher than 37.5°C, we found that the independent risk factors were a higher BMI and a history of not regularly exercising during the past 12 months.

Dehydration is another significant risk factor associated with heat-related illnesses. One of the current gold standards to evaluate hydration status is to measure blood osmolality [46]; however, this method may not be suitable for use in a field setting for numerous soldiers undergoing basic training. To assess dehydration events in the setting of basic military training, we made daily records of body weight and urine color, which are closely related to the hydration status of the trainees [30,47]. Nevertheless, urine specific gravity would be a better option than urine color for evaluating hydration status. This measure is also often used in the field, particularly among athletes. Future research employing urine specific gravity should be evaluated in this new conscript population. Several studies have reported a close relationship between hydration status and the development of heat-related illnesses, particularly heat stroke [48–51]. The magnitude of the increase in core temperature is related to the level of dehydration [2,52]. For the daily body weight assessments, all the participants were measured during the same time period every evening before bed. Weight loss over a short period of time, such as one day, may be explained in part by the direct effects of dehydration [2,45]. In our current study, we used a criterion of more than 3% weight loss in one day to indicate a relatively severe level of dehydration. During the 10-week training period, a loss of more than 3% of body weight in one day occurred 788 times in 809 soldiers. This study demonstrated that a day-to-day decrease in body weight by more than 3% was associated with a high BMI, an indoor occupation and a lack of exercise prior to entering training.

The assessment of urine color is a noninvasive technique that can be utilized by a nonprofessional person to evaluate hydration status [53,54]. Urine color monitoring is widely used among athletes and those who work in high-temperature environments [41,42]. Urine color is normally categorized into several color levels. However, in our current study, we categorized urine color into only 4 groups (light yellow, yellow, brown, and dark brown or darker) to facilitate standardization among various health personnel in the field setting. The trainees’ urine colors were assessed once a day in the evening, and we identified dark brown or darker colored urine 682.11 times/100 person-months, affecting 83.4% of the participants, during the 10-week period.

Previous studies have reported risk factors associated with heat-related illnesses in military populations [36,55,56]. However, our present study reported a relatively expansive set of personal risk factors, i.e., BMI, dehydration and body temperature, and aimed to identify factors suitable for close, continuous monitoring of military recruits during basic training in Thailand. In addition, our major finding of the relatively high rate of dehydration among military recruits during basic training, which is a modifiable factor, may be uniquely beneficial for training in tropical regions and other areas with extremely hot and humid environments.

Personnel overseeing RTA basic training should consider the high rate of dehydration among trainees and improve the water replacement process during training. Improved access to drinking water during training hours may include the use of individual water canteens and allowing the new conscripts to drink water as necessary during the training period [49]. Additionally, the recommended amount of water to be consumed during each training hour, signaled by a colored flag, should be emphasized among the new conscripts.

In addition to daily monitoring for effective prevention, the prevention program should consider screening individual conscripts for known risk factors, particularly BMI status, to identify high-risk subjects at the beginning of the training program and adjust the intensity of the training accordingly. Daily monitoring for personal factors and heat-related illnesses would not only be useful for early detection of risk factors to increase the effectiveness of a prevention program but also increase awareness of the occurrence of heat-related illnesses among the trainers and new conscripts. Such awareness would affect the intensity of physical activities and improve prevention and effective medical care for these patients.

In the present study, we did not demonstrate that daily monitoring of proxy indicators prevented heat-related illnesses. However, we proposed a novel strategy to monitor indicators signaling a high risk for developing heat-related illnesses. Preventive monitoring of risk indicators would identify subjects who are at a high risk of developing heat-related illnesses. These subjects could then be advised to increase their water intake, discontinue training, decrease the intensity of training, or seek medical evaluation. The efficacy of such interventions in this population requires further controlled studies.

In the present study, we focused on some of the major personal risk factors for heat-related illnesses, including BMI, hydration status, and body temperature. However, other risk factors for heat-related illnesses should be considered as well in the design of future monitoring indicators to prevent heat-related illnesses in this population during basic training, including infections [57], illicit drug use [13,45], alcohol consumption [2,45], sleep deprivation [20], a history of heat-related illness [2,18,34], and level of physical fitness [2,18].

This study has several limitations. First, heat-related illnesses were diagnosed daily by training unit medical personnel. Although training sessions on the identification of heat-related illnesses were provided to all medical personnel who participated in this study, active case finding on a regular basis may not have been fully performed during the 10-week period of basic training. This situation might lead to an underestimation of the incidence of heat-related illnesses and a bias toward the null hypothesis for some risk factors in this setting. In terms of daily urine color evaluation, which was categorized into 4 levels, this method is relatively subjective and may not have been uniform across all daily evaluations and training units.

Finally, the validity and reliability of the measurements in our study, including tympanic temperature and body weight measurements, should be considered, and researchers should be made aware of these factors when interpreting this data set. We used the same type of digital thermometer and body weight scale across the study population at every study site to increase the reliability and validity of the measurements. In addition, all of the study personnel had been trained to perform the measurements under the same study protocol across all participating military units.

## Conclusion

Our current study demonstrated that elevated BMI was associated with the occurrence of heat-related illnesses, a decrease in daily body weight by more than 3%, and dark urine color among new RTA conscripts undergoing basic military training. The daily monitoring of heat-related illnesses and other indicators, e.g., tympanic temperature, daily body weight and urine color, was feasible in the field setting. These study results may be used as a basis for developing an additional screening and monitoring program for the prevention of heat-related illnesses among conscripts during basic training in Thailand.

## Supporting information

S1 Table Demographic data. (DOCX)

S2 Table Heat-related outcomes among new conscripts during basic military training. (DOCX)

S3 Table Personal risk factors associated with heat-related illness among new conscripts during basic military training. (DOCX)

S4 Table Personal risk factors associated with a tympanic temperature greater than 37.5°C among new conscripts during basic military training. (DOCX)

S5 Table Personal risk factors associated with body weight loss of more than 3% in one day among new conscripts during basic military training. (DOCX)

S6 Table Personal risk factors associated with the production of dark brown urine by new conscripts during basic military training. (DOCX)

S1 Survey Survey questions used in the study (English translation and Thai versions). (DOCX)

S1 File Database. (XLSX)
