# Supplementary material for: Personal risk factors associated with heat-related illness among new conscripts undergoing basic training in Thailand
Source: PLoS One. 2018 Sep 4;13(9):e0203428. doi: 10.1371/journal.pone.0203428 (PMC6122829; doi:10.1371/journal.pone.0203428)
Supplement: S1 Table — (DOCX) [file pone.0203428.s001.docx]

**Table 1. Demographic Data.**

| **Demographic data** | **Number of study participants (%)** |
| --- | --- |
| **Geographical Army area (n=809)** |  |
| - **Average (min.-max.) temperature and humidity (May-June 2013)** |  |
| Central (Lop Buri province) | 315 (38.9) |
| - Temperature=29.7 ºC (22.8-39.7 ºC) |  |
| - Humidity=70% (28-99%) |  |
| Northern (Chiang Mai province) | 163 (20.2) |
| - Temperature=29.0 ºC (21.8-40.3 °C) |  |
| - Humidity=67% (24-98%) |  |
| Bangkok Metropolitan Region | 136 (16.8) |
| - Temperature=29.6 ºC (23.0-38.8 ºC) |  |
| - Humidity=76% (35-96%) |  |
| North-Eastern (Ubon Ratchathani province) | 129 (15.9) |
| - Temperature=27.7 ºC (20.4-36.8 ºC) |  |
| - Humidity=71% (31-89%) |  |
| Southern (Songkhla province) | 66 (8.2) |
| - Temperature=27.4 ºC (21.7-35.6 ºC) |  |
| - Humidity=83% (45-100%) |  |
| **Age (yrs) – mean±SD** |  |
| **Body mass index (kg/m^2^) (n=743)** |  |
| <18.5 | 76 (10.2) |
| 18.5-22.9 | 434 (58.5) |
| 23.0-24.9 | 96 (12.9) |
| 25.0-29.9 | 96 (12.9) |
| ≥30.0 | 41 (5.5) |
| **Occupation prior to conscription (n=765)** |  |
| Unemployed | 62 (8.1) |
| Student | 115 (15.0) |
| Farmer (farming, gardening, or animals) | 219 (28.6) |
| Employee | 180 (23.5) |
| Laborer | 74 (9.7) |
| Merchant | 51 (6.7) |
| Others | 64 (8.4) |
| **Occupation group prior to conscription (n=701)** |  |
| Indoor (unemployed, student, employee, or merchant) | 408 (58.2) |
| Outdoor (Farmer or Laborer) | 293 (41.8) |
| **Smoking in the past 12 months** |  |
| Current smoker | 516 (67.5) |
| Ex-smoker | 56 (7.3) |
| Never smoked | 193 (25.2) |
| **Exercise in the past 12 months (at least 3 days per week)** |  |
| Yes | 289 (39.8) |
| No | 437 (60.2) |
