# Supplementary material for: Personal risk factors associated with heat-related illness among new conscripts undergoing basic training in Thailand
Source: PLoS One. 2018 Sep 4;13(9):e0203428. doi: 10.1371/journal.pone.0203428 (PMC6122829; doi:10.1371/journal.pone.0203428)
Supplement: S2 Table — (DOCX) [file pone.0203428.s002.docx]

**Table 2. Heat-related Outcomes among New Conscripts during Basic Military Training (n=809).**

| **Heat-related outcomes** | **Number (%)** | **No. of incidents** | **Incidence rate per 100 person-months (95% CI)** |
| --- | --- | --- | --- |
| Heat-related illnesses without prickly heat | 53 (6.6) | 53 | 3.41 (2.55-4.23) |
| Tympanic body temperature greater than 37.5 °C | 104 (12.9) | 136 | 8.27 (7.69-8.39) |
| >37.5 °C-37.9 °C | 66 (8.2) | 89 | 5.41 (4.94-5.87) |
| 38.0 °C-38.9 °C | 24 (3.0) | 29 | 1.76 (1.55-1.97) |
| >39.0 °C | 14 (1.7) | 18 | 1.09 (0.92-1.27) |
| Body weight loss of more than 3% in one day | 463 (57.2) | 788 | 47.91 (44.22-51.58) |
| Dark brown urine | 675 (83.4) | 11220 | 682.11 (635.49-728.52) |
