# Supplementary material for: Personal risk factors associated with heat-related illness among new conscripts undergoing basic training in Thailand
Source: PLoS One. 2018 Sep 4;13(9):e0203428. doi: 10.1371/journal.pone.0203428 (PMC6122829; doi:10.1371/journal.pone.0203428)
Supplement: S3 Table — (DOCX) [file pone.0203428.s003.docx]

**Table 3. Personal Risk Factors Associated with Heat-related Illness among New Conscripts during Basic Military Training.**

| **Personal factors** | **No. of incidents** | | **Incidence rate per 100 person-months** | **Univariate analysis** | | **Multivariate analysis** | |
| --- | --- | --- | --- | --- | --- | --- | --- |
|  |  |  |  | **IRR (95% CI)** | **p-value** | **IRR (95% CI)** | **p-value** |
| **Occupation prior to conscriptions** |  | |  |  |  |  |  |
| Indoor | 24 | | 3.05 | 0.86 (0.47-1.55) | 0.608 |  |  |
| Outdoor | 20 | | 3.56 | 1 |  |  |  |
| **Body mass index (kg/m^2^)** |  | |  |  |  |  |  |
| <18.5 | 7 | | 4.86 | 1.78 (0.77-4.16) | 0.180 | 1.85 (0.79-4.34) | 0.156 |
| 18.5-22.9 | 23 | | 2.72 | 1 |  |  |  |
| 23.0-24.9 | 6 | | 3.27 | 1.20 (0.49-2.95) | 0.691 | 1.23 (0.50-3.05) | 0.648 |
| 25.0-29.9 | 6 | | 3.25 | 1.19 (0.49-2.93) | 0.700 | 1.24 (0.50-3.05) | 0.645 |
| ≥30.0 | 5 | | 6.84 | 2.51 (0.95-6.60) | 0.062 | 2.66 (1.01-7.03) | 0.048 |
| **Smoking in the past 12 months** | | |  |  |  |  |  |
| Current smoker | | 34 | 3.43 | 1.61 (0.75-3.48) | 0.224 | 1.58 (0.73-3.43) | 0.245 |
| Ex-smoker | | 5 | 4.75 | 2.23 (0.73-6.83) | 0.158 | 2.14 (0.70-6.55) | 0.183 |
| Never smoked | | 8 | 2.12 | 1 |  |  |  |
| **Exercise in the past 12 months** | |  |  |  |  |  |  |
| No | | 24 | 2.84 | 0.71 (0.40-1.27) | 0.251 |  |  |
| Yes | | 22 | 3.99 | 1 |  |  |  |
