# Supplementary material for: Personal risk factors associated with heat-related illness among new conscripts undergoing basic training in Thailand
Source: PLoS One. 2018 Sep 4;13(9):e0203428. doi: 10.1371/journal.pone.0203428 (PMC6122829; doi:10.1371/journal.pone.0203428)
Supplement: S4 Table — (DOCX) [file pone.0203428.s004.docx]

**Table 4. Personal Risk Factors Associated with a Tympanic Temperature Greater than 37.5 ºC among New Conscripts during Basic Military Training.**

| **Personal factors** | **No. of incidents** | **Incidence rate per 100 person-months** | **Univariate analysis** | | **Multivariate analysis** | |
| --- | --- | --- | --- | --- | --- | --- |
|  |  |  | **IRR (95% CI)** | **p-value** | **IRR (95% CI)** | **p-value** |
| **Occupation prior to conscriptions** |  |  |  |  |  |  |
| Indoor | 67 | 8.11 | 1.01 (0.69-1.46) | 0.972 |  |  |
| Outdoor | 48 | 8.05 | 1 |  |  |  |
| **Body mass index (kg/m^2^)** |  |  |  |  |  |  |
| <18.5 | 11 | 7.12 | 0.86 (0.46-1.62) | 0.641 | 0.91 (0.48-1.72) | 0.762 |
| 18.5-22.9 | 27 | 8.27 | 1 |  |  |  |
| 23.0-24.9 | 15 | 7.72 | 0.93 (0.54-1.63) | 0.808 | 1.04 (0.60-1.83) | 0.882 |
| 25.0-29.9 | 19 | 9.83 | 1.19 (0.72-1.97) | 0.502 | 1.32 (0.79-2.19) | 0.293 |
| ≥30.0 | 8 | 9.68 | 1.17 (0.56-2.43) | 0.672 | 1.22 (0.58-2.53) | 0.602 |
| **Smoking in the past 12 months** |  |  |  |  |  |  |
| Current smoker | 84 | 8.02 | 0.98 (0.65-1.47) | 0.907 |  |  |
| Ex-smoker | 11 | 9.67 | 1.18 (0.59-2.34) | 0.638 |  |  |
| Never smoked | 32 | 8.21 | 1 |  |  |  |
| **Exercise in the past 12 months** |  |  |  |  |  |  |
| No | 84 | 9.50 | 1.51 (1.03-2.22) | 0.037 | 1.62 (1.09-2.40) | 0.017 |
| Yes | 37 | 6.29 | 1 |  |  |  |
