# Supplementary material for: Personal risk factors associated with heat-related illness among new conscripts undergoing basic training in Thailand
Source: PLoS One. 2018 Sep 4;13(9):e0203428. doi: 10.1371/journal.pone.0203428 (PMC6122829; doi:10.1371/journal.pone.0203428)
Supplement: S5 Table — (DOCX) [file pone.0203428.s005.docx]

**Table 5. Personal Risk Factors Associated with Body Weight Loss of More than 3% in One Day among New Conscripts during Basic Military Training.**

| **Personal factors** | **No. of incidents** | **Incidence rate per 100 person-months** | **Univariate analysis** | | **Multivariate analysis** | |
| --- | --- | --- | --- | --- | --- | --- |
|  |  |  | **IRR (95% CI)** | **p-value** | **IRR (95% CI)** | **p-value** |
| **Occupation prior to conscription** |  |  |  |  |  |  |
| Indoor | 446 | 54.00 | 1.33 (1.14-1.55) | <0.001 | 1.33 (1.12-1.58) | 0.001 |
| Outdoor | 242 | 40.63 |  |  |  |  |
| **Body mass index (kg/m^2^)** |  |  |  |  |  |  |
| <18.5 | 76 | 49.16 | 1.19 (0.93-1.53) | 0.158 | 1.18 (0.91-1.53) | 0.218 |
| 18.5-22.9 | 434 | 41.14 | 1 |  |  |  |
| 23.0-24.9 | 96 | 54.06 | 1.31 (1.06-1.63) | 0.014 | 1.30 (1.02-1.64) | 0.030 |
| 25.0-29.9 | 96 | 59.52 | 1.45 (1.17-1.78) | 0.001 | 1.47 (1.17-1.83) | 0.001 |
| ≥30.0 | 41 | 64.17 | 1.56 (1.17-2.08) | 0.003 | 1.69 (1.23-2.33) | 0.001 |
| **Smoking in the past 12 months** |  |  |  |  |  |  |
| Current smoker | 516 | 45.71 | 0.93 (0.79-1.10) | 0.414 |  |  |
| Ex-smoker | 56 | 58.95 | 1.20 (0.91-1.59) | 0.194 |  |  |
| Never smoked | 193 | 49.02 | 1 |  |  |  |
| **Exercise in the past 12 months** |  |  |  |  |  |  |
| No | 398 | 45.02 | 0.86 (0.74-1.00) | 0.046 | 0.83 (0.71-0.98) | 0.023 |
| Yes | 308 | 52.38 | 1 |  |  |  |
