# Supplementary material for: Personal risk factors associated with heat-related illness among new conscripts undergoing basic training in Thailand
Source: PLoS One. 2018 Sep 4;13(9):e0203428. doi: 10.1371/journal.pone.0203428 (PMC6122829; doi:10.1371/journal.pone.0203428)
Supplement: S6 Table — (DOCX) [file pone.0203428.s006.docx]

**Table 6. Personal Risk Factors Associated with the Production of Dark Brown Urine by New Conscripts during Basic Military Training.**

| **Personal risk factors** | **No. of incidents** | **Incidence rate per 100 person-months)** | **Univariate analysis** | | **Multivariate analysis** | |
| --- | --- | --- | --- | --- | --- | --- |
|  |  |  | **IRR (95% CI)** | **p-value** | **IRR (95% CI)** | **p-value** |
| **Occupation prior to conscriptions** |  |  |  |  |  |  |
| Indoor | 5630 | 681.67 | 1.05 (1.01-1.09) | 0.022 | 1.00 (0.96-1.05) | 0.945 |
| Outdoor | 3870 | 649.79 | 1 |  |  |  |
| **Body mass index (kg/m^2^)** |  |  |  |  |  |  |
| <18.5 | 1191 | 771.24 | 1.29 (1.21-1.38) | <0.001 | 1.23 (1.14-1.32) | <0.001 |
| 18.5-22.9 | 5264 | 596.61 | 1 |  |  |  |
| 23.0-24.9 | 1458 | 751.65 | 1.26 (1.19-1.33) | <0.001 | 1.30 (1.22-1.38) | <0.001 |
| 25.0-29.9 | 1890 | 978.19 | 1.64 (1.56-1.73) | <0.001 | 1.65 (1.56-1.75) | <0.001 |
| ≥30.0 | 873 | 1057.03 | 1.77 (1.65-1.90) | <0.001 | 1.71 (1.57-1.86) | <0.001 |
| **Smoking in the past 12 months** |  |  |  |  |  |  |
| Current smoker | 996 | 763.04 | 1.42 (1.35-1.49) | <0.001 | 1.50 (1.42-1.58) | <0.001 |
| Ex-smoker | 747 | 657.34 | 1.22 (1.12-1.33) | <0.001 | 1.17 (1.07-1.28) | 0.001 |
| Never smoked | 97 | 538.19 | 1 |  |  |  |
| **Exercise in the past 12 months** |  |  |  |  |  |  |
| No | 6474 | 732.32 | 1.11 (1.07-1.16) | <0.001 | 1.10 (1.05-1.15) | <0.001 |
| Yes | 3877 | 659.39 | 1 |  |  |  |
