# Supplementary material for: Personal risk factors associated with heat-related illness among new conscripts undergoing basic training in Thailand
Source: PLoS One. 2018 Sep 4;13(9):e0203428. doi: 10.1371/journal.pone.0203428 (PMC6122829; doi:10.1371/journal.pone.0203428)
Supplement: S1 Survey — (DOCX) [file pone.0203428.s007.docx]

**Survey Questions Used in the Study (English Translation)**

| **FORM B** | | **Training Unit Basic Information** | | | | | |
| --- | --- | --- | --- | --- | --- | --- | --- |
| **DATE** ⬜⬜/⬜⬜/⬜⬜⬜⬜ (dd/mm/yyyy) | | | | **Unit ID**  ⬜⬜⬜⬜⬜⬜ | | | |
| **Environment data** | **Lists** | | **Time period** | | | | |
|  |  |  | **06.00-08.59** | | **09.00-11.59** | **12.00-14.59** | **15.00-17.00** |
| **Flag Color** | 1. White (W) | | **❑** | | **❑** | **❑** | **❑** |
|  | 1. Green (G) | | **❑** | | **❑** | **❑** | **❑** |
|  | 1. Yellow (Y) | | **❑** | | **❑** | **❑** | **❑** |
|  | 1. Red (R) | | **❑** | | **❑** | **❑** | **❑** |
|  | 1. Black (B) | | **❑** | | **❑** | **❑** | **❑** |
| **Temperature** | 1) Dry bulb temperature (^๐^C)T1 | | ⬜⬜⬜ | | ⬜⬜⬜ | ⬜⬜⬜ | ⬜⬜⬜ |
|  | 2) Wet bulb temperature (^๐^C)T2 | | ⬜⬜⬜ | | ⬜⬜⬜ | ⬜⬜⬜ | ⬜⬜⬜ |

|  |  | | **Form C** | | **Personal Information**  **ID** ⬜⬜⬜⬜⬜⬜-⬜⬜⬜ |
| --- | --- | --- | --- | --- | --- |
| 1. Name | |  | | | |
| 2. Date of Birth | | ⬜⬜/⬜⬜/⬜⬜⬜⬜ (dd/mm/yyyy) | | | |
| 3. Age | | ⬜⬜ years | | | |
| 4. Body Weight ⬜⬜⬜.⬜ Kg. | | | | 5. Height ⬜⬜⬜.⬜ Centimetres | |
| 6. Name of training unit | | | |  | |
| 7. Occupation prior to induction  **❑** 1. None  **❑** 6. Employer  **❑** 2. Student **❑** 7. Laborer  **❑** 3. Rice field Farmer  **❑** 8. Merchant  **❑** 4. Plantation Farmer  **❑** 9. Other, Please specify ………  **❑** 5. Poultry Farmer | | | | | |
| 8. History of Smoking during the past 12 months  **❑** 1. Current Smoker, Please specify the amount of cigarette  **❑** 2. Ex-smoker  **❑** 3. Non smoker | | | | | |
| 9. During the past 12 months prior to induction into the army, have you regularly exercise for an average of 20-30 minutes per session at least 3 sessions per week?  **❑** 1. Yes Please specify number of days per week ⬜ days/week  **❑** 2. No | | | | | |

|  | | **FORM D Daily Personal Record**  **ID** ⬜⬜⬜⬜⬜⬜-⬜⬜⬜ |
| --- | --- | --- |
| **Unit ID** : ⬜⬜⬜⬜⬜⬜ | **DD/MM/YYYY** : ⬜⬜/⬜⬜/⬜⬜⬜⬜ | |

| **No.** | **ID** | **1. Body Temp.** | **2. Body Weight** | **3. Signs and Symptoms** | | | | | | |
| --- | --- | --- | --- | --- | --- | --- | --- | --- | --- | --- |
|  |  |  |  | **D1** | **D2** | **D3** | **D4** | **D5** | **D6** | **D7** |
|  | ⬜⬜⬜ | ⬜⬜.⬜ | ⬜⬜⬜.⬜ | **❑** | **❑** | **❑** | **❑** | **❑** | **❑** | **❑** |
|  |  |  |  | **❑ D11**  **❑ D12**  **❑ D13**  **❑ D14** |  |  |  |  |  |  |
|  | ⬜⬜⬜ | ⬜⬜.⬜ | ⬜⬜⬜.⬜ | **❑** | **❑** | **❑** | **❑** | **❑** | **❑** | **❑** |
|  |  |  |  | **❑ D11**  **❑ D12**  **❑ D13**  **❑ D14** |  |  |  |  |  |  |
|  | ⬜⬜⬜ | ⬜⬜.⬜ | ⬜⬜⬜.⬜ | **❑** | **❑** | **❑** | **❑** | **❑** | **❑** | **❑** |
|  |  |  |  | **❑ D11**  **❑ D12**  **❑ D13**  **❑ D14** |  |  |  |  |  |  |
|  | ⬜⬜⬜ | ⬜⬜.⬜ | ⬜⬜⬜.⬜ | **❑** | **❑** | **❑** | **❑** | **❑** | **❑** | **❑** |
|  |  |  |  | **❑ D11**  **❑ D12**  **❑ D13**  **❑ D14** |  |  |  |  |  |  |
|  | ⬜⬜⬜ | ⬜⬜.⬜ | ⬜⬜⬜.⬜ | **❑** | **❑** | **❑** | **❑** | **❑** | **❑** | **❑** |
|  |  |  |  | **❑ D11**  **❑ D12**  **❑ D13**  **❑ D14** |  |  |  |  |  |  |
|  | ⬜⬜⬜ | ⬜⬜.⬜ | ⬜⬜⬜.⬜ | **❑** | **❑** | **❑** | **❑** | **❑** | **❑** | **❑** |
|  |  |  |  | **❑ D11**  **❑ D12**  **❑ D13**  **❑ D14** |  |  |  |  |  |  |

**FORM D Daily Personal Record**

**1. Body Weight**  ⬜⬜⬜.⬜ Kilograms

**2. Body Temperature** ⬜⬜.⬜ Degree Celsius

**3. Daily Sings and Symptoms**

**D1** 🡆 3.1 **Urine Color**

D11 🢧 1. Dark Brown D12 🢧 2. Dark Yellow

D13 🢧 3. Yellow D14 🢧 4. Light Yellow

**D2** 🡆 **3.2 Heat edema**

**D3 🡆 3.3 Heat Rash/ Prickly Heat**

**D4** 🡆 **3.4 Heat Syncope**

**D5** 🡆 3.5 **Heat cramps**

**D6** 🡆 3.6 **Heat tetany**

**D7** 🡆 **3.7 Heat exhaustion**

**Survey Questions Used in the Study (Thai Version))**

| **แบบบันทึก ข** | | | **ข้อมูลพื้นฐาน หน่วยฝึก** | | | | | |
| --- | --- | --- | --- | --- | --- | --- | --- | --- |
| **วัน** เดือน ปี ( / / ) | | | | | **รหัส**  ⬜⬜⬜⬜⬜⬜ | | | |
| **ข้อมูลสิ่งแวดล้อม** | **รายการ** | | | **ระยะเวลา** | | | | |
|  |  |  |  | **06.00-08.59** | | **09.00-11.59** | **12.00-14.59** | **15.00-17.00** |
| ธงสัญญาณ | | 1. ธงขาว (W) | | **❑** | | **❑** | **❑** | **❑** |
|  |  | 1. ธงเขียว (G) | | **❑** | | **❑** | **❑** | **❑** |
|  |  | 1. ธงเหลือง(Y) | | **❑** | | **❑** | **❑** | **❑** |
|  |  | 1. ธงแดง(R) | | **❑** | | **❑** | **❑** | **❑** |
|  |  | 1. ธงดำ (B) | | **❑** | | **❑** | **❑** | **❑** |
| **อุณหภูมิ** | | 1) อุณหภูมิกระเปาะแห้ง (^๐^C)T1 | | ⬜⬜⬜ | | ⬜⬜⬜ | ⬜⬜⬜ | ⬜⬜⬜ |
|  |  | 2) อุณหภูมิกระเปาะเปียก (^๐^C)T2 | | ⬜⬜⬜ | | ⬜⬜⬜ | ⬜⬜⬜ | ⬜⬜⬜ |

|  | **แบบบันทึก ค** | | **ข้อมูลส่วนบุคคลพื้นฐาน**  รหัสตัวอย่าง ⬜⬜⬜⬜⬜⬜-⬜⬜⬜ |
| --- | --- | --- | --- |
| 1. ชื่อ-สกุล |  | | |
| 2. วัน เดือน ปี เกิด | ⬜⬜/⬜⬜/⬜⬜⬜⬜ | | |
| 3. อายุ | ⬜⬜ ปี | | |
| 4. น้ำหนัก ⬜⬜⬜.⬜ กิโลกรัม | | 5. ส่วนสูง ⬜⬜⬜.⬜ เซนติเมตร | |
| 6. ชื่อหน่วยฝึก |  |  |  |
|  |  |  |  |
|  |  |  |  |
| 7. อาชีพล่าสุดก่อนมาเป็นทหาร  **❑** 1. ไม่มีอาชีพ อยู่บ้านเฉยๆ **❑** 6. พนักงานบริษัท **❑** 2. นักเรียน นักศึกษา  **❑** 7. ผู้ใช้แรงงาน/ก่อสร้าง  **❑** 3. ทำนา ทำไร่ (รวมทั้ง รับจ้างทำ/ทำเอง) **❑** 8. ค้าขาย  **❑** 4. ทำสวน  **❑** 9. อื่นๆระบุ…………  **❑** 5. เลี้ยงสัตว์/ปศุสัตว์ | | | |
| 8. ประวัติการสูบบุหรี่ ใน 12 เดือนที่ผ่านมา  **❑** 1. สูบ และปัจจุบันยังสูบอยู่  **❑** 2. เคยสูบ แต่ปัจจุบันเลิกแล้ว  **❑** 3. ไม่เคยสูบเลย | | | |
| 9. ประวัติการออกกำลังกาย ใน 12 เดือนที่ผ่านมาก่อนเป็นทหาร ได้ออกกำลังกายเฉลี่ย 20-30 นาทีต่อครั้ง อย่างน้อย 3 ต่อสัปดาห์ หรือไม่   1. ได้ออกกำลังกาย : เฉลี่ย..............วัน/สัปดาห์   2. ไม่ได้ออกกำลังกาย | | | |

|  | | **แบบฟอร์ม ง ข้อมูลส่วนบุคคลรายวัน** |
| --- | --- | --- |
| **รหัสตัวอย่าง : ⬜⬜⬜⬜⬜⬜** | **วัน เดือน ปี : ⬜⬜/⬜⬜/⬜⬜⬜⬜** | |

| **ลำดับ** | **รหัส** | **1. อุณหภูมิร่างกาย.** | **2. น้ำหนักตัว** | **3. อาการและอาการแสดง** | | | | | | |
| --- | --- | --- | --- | --- | --- | --- | --- | --- | --- | --- |
|  |  |  |  | **D1** | **D2** | **D3** | **D4** | **D5** | **D6** | **D7** |
|  | ⬜⬜⬜ | ⬜⬜.⬜ | ⬜⬜⬜.⬜ | **❑** | **❑** | **❑** | **❑** | **❑** | **❑** | **❑** |
|  |  |  |  | **❑ D11**  **❑ D12**  **❑ D13**  **❑ D14** |  |  |  |  |  |  |
|  | ⬜⬜⬜ | ⬜⬜.⬜ | ⬜⬜⬜.⬜ | **❑** | **❑** | **❑** | **❑** | **❑** | **❑** | **❑** |
|  |  |  |  | **❑ D11**  **❑ D12**  **❑ D13**  **❑ D14** |  |  |  |  |  |  |
|  | ⬜⬜⬜ | ⬜⬜.⬜ | ⬜⬜⬜.⬜ | **❑** | **❑** | **❑** | **❑** | **❑** | **❑** | **❑** |
|  |  |  |  | **❑ D11**  **❑ D12**  **❑ D13**  **❑ D14** |  |  |  |  |  |  |
|  | ⬜⬜⬜ | ⬜⬜.⬜ | ⬜⬜⬜.⬜ | **❑** | **❑** | **❑** | **❑** | **❑** | **❑** | **❑** |
|  |  |  |  | **❑ D11**  **❑ D12**  **❑ D13**  **❑ D14** |  |  |  |  |  |  |
|  | ⬜⬜⬜ | ⬜⬜.⬜ | ⬜⬜⬜.⬜ | **❑** | **❑** | **❑** | **❑** | **❑** | **❑** | **❑** |
|  |  |  |  | **❑ D11**  **❑ D12**  **❑ D13**  **❑ D14** |  |  |  |  |  |  |
|  | ⬜⬜⬜ | ⬜⬜.⬜ | ⬜⬜⬜.⬜ | **❑** | **❑** | **❑** | **❑** | **❑** | **❑** | **❑** |
|  |  |  |  | **❑ D11**  **❑ D12**  **❑ D13**  **❑ D14** |  |  |  |  |  |  |

**แบบฟอร์ม ง ข้อมูลส่วนบุคคลรายวัน**

1. น้ำหนักตัว ⬜⬜⬜.⬜ กิโลกรัม

2. อุณหภูมิร่างกาย ⬜⬜.⬜ องศาเซลเซียส

3. อาการและอาการแสดง

D1 🡆 3.1 สีปัสสาวะ

D11 🢧 1. สีโค้ก D12 🢧 2. สีเหลืองเข้ม

D13 🢧 3. สีเหลือง D14 🢧 4. สีเหลืองอ่อน

D2 🡆 3.2 การบวมแดด

D3 🡆 3.3 ผดผื่นคันจากความร้อน

D4 🡆 3.4 ลมแดด

D5 🡆 3.5 ตะคริวแดด

D6 🡆 3.6 การเกร็งแดด

D7 🡆 3.7 การเพลียแดด
